# Virtual orthodontic setup in orthodontic camouflage planning for skeletal Class III malocclusion

**DOI:** 10.1590/2177-6709.23.2.075-086.bbo

**Published:** 2018

**Authors:** Felipe Augusto M. Barreto, João Roberto R. da Costa Santos

**Affiliations:** 1 Private practice (Aracaju/SE, Brazil).; 2 Universidade Tiradentes, Post-graduate Course in Orthodontics (Aracaju/SE, Brazil).

**Keywords:** Orthodontics, corrective, Crossbite, Tooth, supernumerary, Incisor, Tooth extraction.

## Abstract

The purpose of this paper was to emphasize the importance of the orthodontic setup in treatment planning for skeletal Class III malocclusion correction in an adult patient with moderate lower anterior crowding and anterior crossbite associated with two supernumerary lower incisors.

## RESUMO

O objetivo desse artigo é enfatizar a importância do setup ortodôntico no planejamento do tratamento da má oclusão de Classe III esquelética de uma paciente adulta com apinhamento anteroinferior moderado e mordida cruzada anterior associada à presença de dois incisivos inferiores supranumerários.

## INTRODUCTION

The treatment of skeletal Class III malocclusion in adult patients is a challenge for the orthodontist, mainly in the choice between compensatory orthodontic treatment or orthodontic preparation for orthognathic surgery.^1^ ^-^ ^7^

In many situations, the fear of the surgical procedure associated with the patient’s satisfaction with their facial appearance makes orthodontic camouflage the patient’s treatment of choice.^1^ ^,^ ^4^ ^,^ ^6^ ^,^ ^8^ Thus, orthodontic treatment associated with tooth extractions is an approach for orthodontic compensation in patients with mild or moderate skeletal Class III malocclusion.^6^ ^,^ ^8^ ^,^ ^9^ ^,^ ^10^ Traditionally, premolars are the most indicated teeth for extraction with camouflage purposes; however, other extraction alternatives have been used, such as extraction of a lower incisor.^3^ ^,^ ^5^ ^,^ ^6^ ^,^ ^9^ ^,^ ^11^

The extraction of a lower incisor in Class III treatment is an option primarily used in patients with Bolton’s tooth-size discrepancy.^5^ The presence of this discrepancy significantly influences orthodontic planning, requiring reduction of dental mass, either by interproximal stripping or by tooth extraction.^12^ ^,^ ^13^

The presence of supernumerary teeth promotes upper and lower dental disproportion. This disharmony may lead to malocclusions and difficulties in obtaining adequate overjet and overbite.^12^ ^-^ ^15^ The prevalence of supernumerary teeth in the anterior mandibular region is very low and varies according to the studied population.^16^ ^,^ ^17^ Therefore, the presence of six lower incisors becomes an extremely rare clinical condition.

The extraction of a lower incisor provides an increase in overjet and overbite, a desirable effect in Class III patients.^10^ However, prudence and caution are essential for the good orthodontist. Thus, an orthodontic setup is necessary to allow visualization of the final occlusion and to determine how many and which teeth should be extracted.^5^ ^,^ ^10^ ^,^ ^18^ ^,^ ^19^

Thus, the present article describes the treatment of an adult patient presenting skeletal Class III malocclusion with moderate lower anterior crowding and anterior crossbite associated with the presence of two supernumerary lower incisors.

## CLINICAL CASE REPORT

Brown-skinned patient, 20 years old, female, presented with good general health and excellent oral health. Her main complaint concerned the projection of the lower incisors out of the mouth. She presented a Class III skeletal pattern, aggravated by the lack of space for correct alignment of the lower arch, due to the presence of two supernumerary lower incisors.

Frontal facial examination revealed mandibular asymmetry to the right side. In sagittal view, the lower facial third was increased in comparison to the upper and middle thirds. The facial profile was concave due to mandibular projection, with passive lip seal. The aesthetics of smile was impaired due to the anterior crossbite.

**Figure 1:**
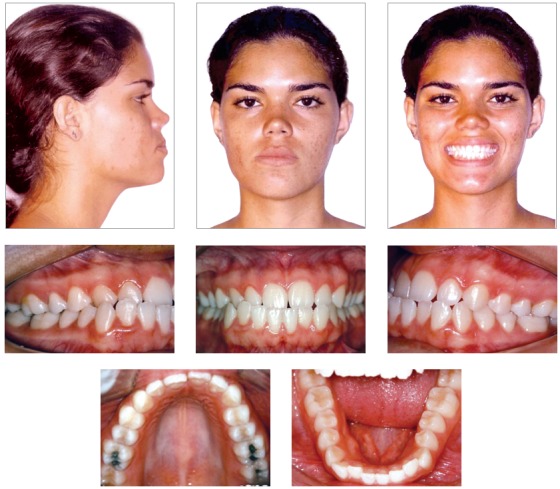
Initial facial and intraoral photographs.

The patient had a Class III skeletal pattern, and facial growth was predominantly horizontal. Occlusal analysis revealed Angle Class I malocclusion with 1-mm overbite and anterior crossbite. The mandibular arch presented moderate anterior crowding and the maxillary arch exhibited anterior contraction on the right side (Fig 1).

Bolton’s analysis revealed inferior excess of 7.5 mm, considering the proportion between the sum of the mesiodistal widths of the fourteen lower and twelve upper teeth; and inferior excess of 9.2 mm, considering the proportion between the anterior lower teeth with the anterior upper teeth.

**Figure 2:**
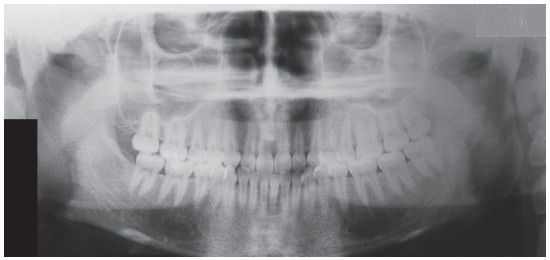
Initial panoramic radiograph.

**Figure 3:**
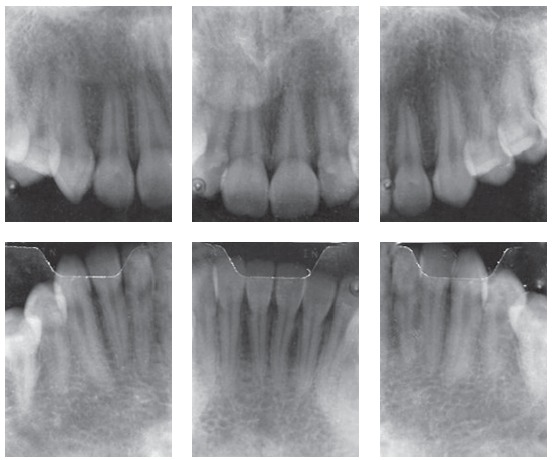
Initial periapical radiographs.

The periapical and panoramic radiographs revealed intact roots, absence of the upper and lower third molars on the right side, presence of two fully erupted supernumerary incisors, as well as light horizontal bone loss in the lower arch (Figs 2 and 3).

**Figure 4:**
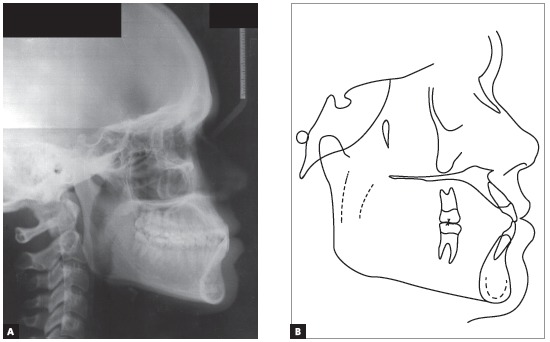
Initial lateral cephalometric radiograph (A) and cephalometric tracing (B).

Cephalometry confirmed the Class III skeletal pattern with ANB = -3^o^, horizontal growth pattern (SN.GoGn = 22^o^ and FMA = 12^o^) and compensatory inclinations of the incisors (1.NA = 29^o^, 1.NB = 37^o^ and IMPA = 107^o^).This positioning of incisors contributed to an unfavorable tegumentary relationship that impaired the patient’s facial aesthetics (Upper lip - S line = 0mm, Lower lip - S line = 4mm) (Table 1 and Fig. 4).

**Table 1:** Cephalometric values: A) initial, B) final and C) two years after the end of orthodontic treatment.

	Measurements		Normal	A	B	Dif. A/B	C
Skeletal pattern	SNA	(Steiner)	82^o^	85^o^	85^o^	0	86^o^
	SNB	(Steiner)	80^o^	88^o^	87^o^	1	88^o^
	ANB	(Steiner)	2^o^	-3^o^	-2^o^	1	-2^o^
	Angle of convexity	(Downs)	0^o^	-6^o^	-5^o^	1	-4^o^
	Y-axis	(Downs)	59^o^	52^o^	52^o^	0	52^o^
	Facial angle	(Downs)	87^o^	98^o^	97^o^	1	96^o^
	SN-GoGn	(Steiner)	32^o^	22^o^	22^o^	0	22^o^
	FMA	(Tweed)	25^o^	12^o^	13^o^	1	14^o^
Dental pattern	IMPA	(Tweed)	90^o^	107^o^	97^o^	10	95^o^
	1.NA (degrees)	(Steiner)	22^o^	29^o^	30^o^	1	29^o^
	1-NA (mm)	(Steiner)	4 mm	14mm	12mm	2	11mm
	1.NB (degrees)	(Steiner)	25^o^	37^o^	25^o^	12	25^o^
	1-NB (mm)	(Steiner)	4 mm	8mm	8mm	0	6mm
	- Interincisal angle	(Downs)	130^o^	119^o^	128^o^	9	128^o^
	1-APo	(Ricketts)	1 mm	1mm	2mm	1	1mm
Profile	Upper lip - S-line	(Steiner)	0 mm	0mm	2mm	2	1mm
	Lower lip - S-line	(Steiner)	0 mm	4mm	3mm	1	1mm

## TREATMENT PLAN AND MECHANICS EMPLOYED

**Figure 5:**
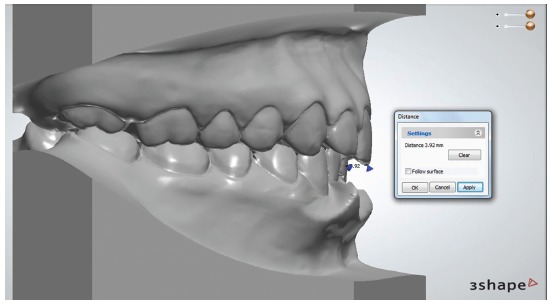
Virtual setup with excessive overjet quantification.

**Figure 6:**
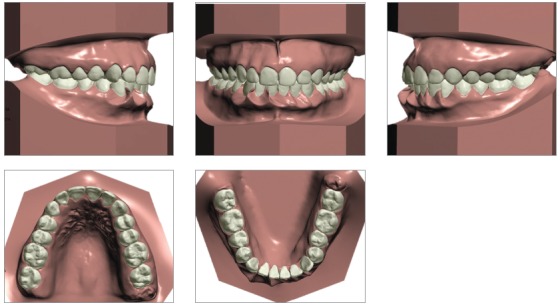
Virtual setup simulating the extraction of two lower incisors.

Considering that the patient discarded the possibility of orthodontic-surgical treatment, the orthodontic planning consisted in correcting Bolton’s tooth-size discrepancy at the anterior region to obtain a harmonious relationship between the dental arches. The virtual orthodontic setup was performed with extraction of two lower incisors, which revealed an unsatisfactory occlusal result, with excessive overjet of 3.92 mm (Figs 5 and 6). Therefore, it was decided to extract only one supernumerary incisor, adjacent to the lower right canine, associated with interproximal 3-mm stripping, distributed among the five remaining incisors. It was verified on the orthodontic setup that this strategy would be sufficient to balance the tooth-size discrepancy between the upper and lower arches, to dissolve the lower anterior crowding and to correct the anterior crossbite.

Treatment was started by bonding Edgewise brackets, 0.022 x 0.028-in slot, using 0.015-in Twist Flex arches for alignment and leveling and 0.014-in, 0.016-in, 0.018-in stainless steel arches for projection of upper incisors and retraction of lower incisors, to aid the correction of the negative overjet. After extraction of the supernumerary incisor, lingual traction of the right lower canine was started, with a chain elastic anchored at a button bonded to its lingual surface and connected to a hook welded in the previously placed Nance lingual arch. In the posterior region, occlusal stops made of self-curing resin (Triad VLC, Dentsply GAC) were added bilaterally, causing anterior disocclusion to aid uncrossing the bite. After closing the extraction space and removing the occlusal stops, it was verified that the incisors presented an edge-to-edge relationship. The planned interproximal stripping was performed and the spaces were closed with active tie-backs in the 0.018-in arch with lingual traction, since new hooks were welded to the lingual arch and buttons were bonded to the lingual surface of the lower incisors. Class III intermaxillary elastics (5/16-in) were used under protocol of continuous use and daily exchanges, thus obtaining positive overjet. Orthodontic finalization was performed using round arches, without the need of incorporating torques into the teeth.

After treatment completion, a removable upper Hawley retainer was used as well as a lingual retainer, made with 0.032-in Twist Flex, bonded to the lower canines.

## RESULTS

**Figure 7:**
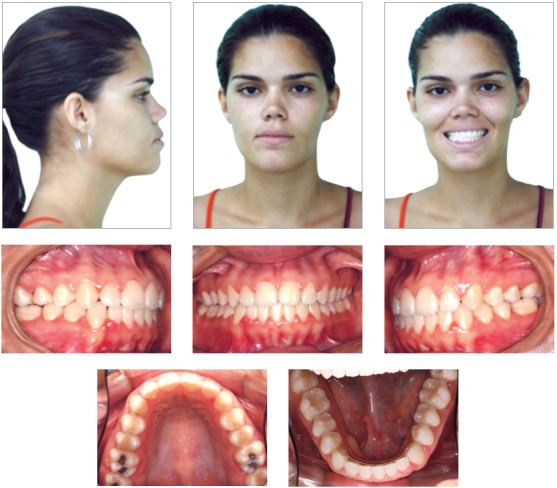
Final facial and intraoral photographs.

At treatment completion, the facial aesthetics was improved and, although five lower incisors were maintained, normal overbite and overjet were obtained, leading canines and molars to Angle Class I, upper midline coinciding with the center of the central lower incisors, resulting in anterior guidance and right and left functional laterality, providing masticatory efficiency and TMJ integrity (Fig 7).

The most marked effects were observed in the lower arch, in which extraction of only one supernumerary incisor and interproximal stripping corrected the anterior crossbite and crowding, with better positioning of teeth in the bone base.

Bolton’s analysis of the final models revealed correction of the discrepancy between the sum of mesiodistal diameters of the thirteen lower teeth and twelve upper teeth, with reduction of the anterior discrepancy to 1.0 mm.

**Figure 8:**
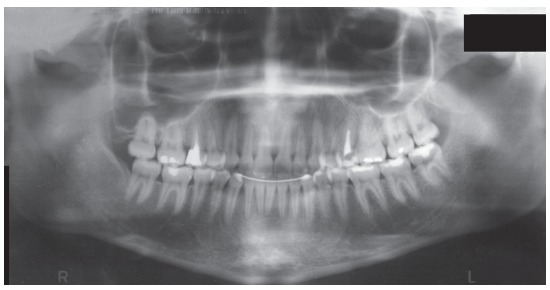
Final panoramic radiograph.

The final panoramic radiograph shows preservation of root lengths and integrity of the alveolar bone crests - except for the mandibular incisor that finished centralized with the upper midline, good root parallelism was observed (Fig 8).

**Figure 9:**
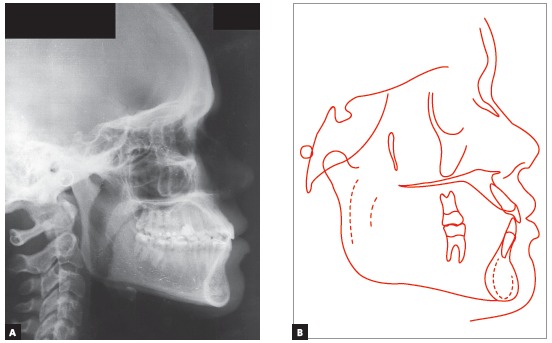
Final lateral cephalometric radiograph (A) and cephalometric tracing (B).

Since the treatment comprised dental compensation, there were no changes in maxillary and mandibular positioning. The patient continued with a Class III skeletal pattern, ANB equal to -2^o^ (SNA = 85^o^ and SN.GoGn = 87^o^), and maintained the horizontal pattern (SN.GoGn = 22^o^ and FMA = 13^o^). However, the patient’s facial profile became more harmonious due to the better relation between the upper and lower lips (Upper lip - S line = 2mm and Lower lip - S line = 3mm). In addition, there was evident decrease in the inclination of mandibular incisors and a small increase in the labial inclination of upper incisors, which allowed correction of the anterior crossbite (1.NA from 29^o^ to 30^o^ and 1.NB from 37^o^ to 25^o^) (Fig 9).

## DISCUSSION

The orthodontic compensatory treatment of a skeletal malocclusion is one of the possible alternatives for adult patients who choose not to undergo surgical procedures^1^ ^,^ ^4^ ^,^ ^8^. In the case reported, orthognathic surgery was discarded in response to the patient’s desire, since there were no complaints regarding facial esthetics and the skeletal discrepancy was small. The patient had hyperdontia, an anomaly related to the presence of supernumerary teeth, whose etiology may be related to heredity.^17^ ^,^ ^20^ ^,^ ^21^ ^,^ ^22^ However, in the present case there were no reports of supernumerary teeth in the family. Hyperdontia affects more males, at a 2:1 ratio,^17^ ^,^ ^20^ ^,^ ^21^ ^,^ ^22^ presenting more frequently as a single supernumerary tooth, with strong predilection for the maxillary anterior region.^16^ ^,^ ^17^ ^,^ ^20^ ^,^ ^22^ In contrast, in the case reported, the patient is female and had two supernumerary teeth in the mandibular anterior region.

Although the presence of supernumerary teeth precludes the achievement of occlusion with normal overjet and overbite,^12^ ^-^ ^15^ the patient always had molars and canines in Class I relation, since the tooth-size excess was located at the incisors region.

The extraction of a lower incisor is a therapeutic alternative for adult patients with mild to moderate Class III malocclusion, which does not fit in the conventional options of extractions.^18^ ^,^ ^19^ In the present case, the decision to extract only one supernumerary lower incisor was determined by the virtual orthodontic setup, which evidenced that it was not necessary to extract two supernumerary incisors to solve the Bolton’s tooth-size discrepancy between the upper and lower arches. With extraction of both supernumerary incisors, Bolton’s analysis showed a superior excess of 3.8 mm; and 2.5 mm considering only the six anterior teeth.

The extraction of the supernumerary tooth adjacent to the right lower canine was indicated because this tooth was closer to the malocclusion region. Reduction of the mesiodistal diameter by interproximal stripping of the five lower incisors provided balance between the dental masses, allowing the overjet correction. Determination of the required amount of interproximal stripping was also based on the Bolton’s analysis of the initial models, which pointed to a tooth size excess of 3 mm in the lower anterior region, even after extracting the supernumerary tooth adjacent to the right lower canine.

**Figure 10:**
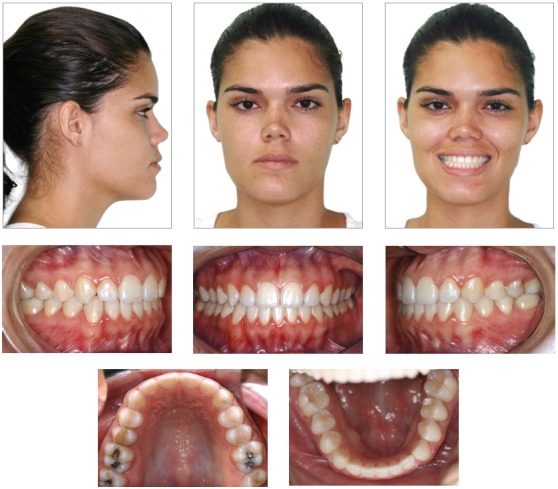
Facial and intraoral photographs two years after treatment completion.

**Figure 11:**
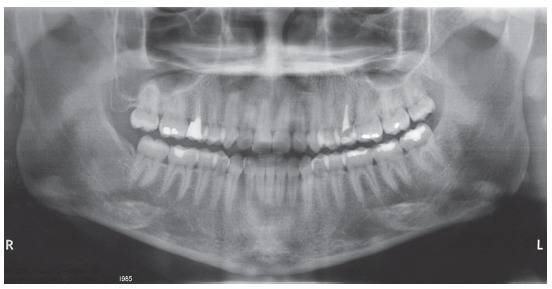
Panoramic radiography two years after treatment completion.

**Figure 12:**
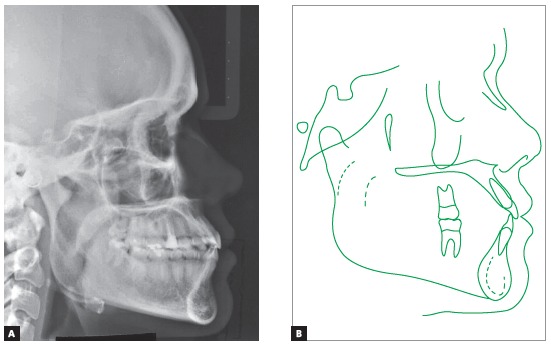
Lateral cephalometric radiograph (A) and cephalometric tracing (B) two years after treatment completion.

Cases treated with extraction of a lower incisor present a lower rate of post-treatment crowding relapse, due to maintenance or reduction of intercanine distance.^18^ ^,^ ^19^ This stability can be clinically and radiographically demonstrated after two years of treatment completion in the present case, as shown in Figures 10, 11 and 12.

**Figure 13:**
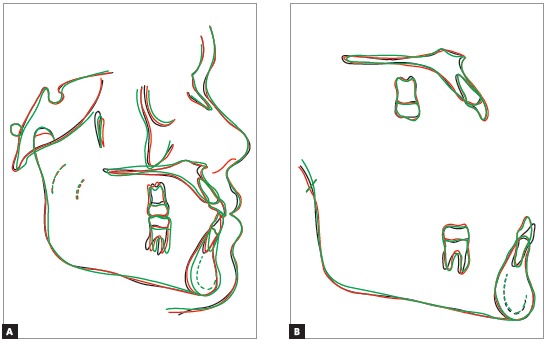
Total (A) and partial (B) superimpositions of cephalometric tracings obtained at treatment onset, completion and two years after treatment completion.

Tracings superimposition shows decrease of the profile’s concavity because the upper lip acquired an anterior position, improving its relationship with the lower lip (Fig 13).

## CONCLUSIONS

Bolton’s discrepancy analysis and virtual orthodontic setup are important diagnostic tools that assist in the planning of atypical cases, such as the present one. Compensatory orthodontic treatment of mild to moderate skeletal Class III with extraction of lower incisor is an effective therapeutic possibility that should be considered by orthodontists, but with careful and appropriate planning.
